# Supplementary material for: The Interactive Effects of Severe Vitamin D Deficiency and Iodine Nutrition Status on the Risk of Thyroid Disorder in Pregnant Women
Source: Nutrients. 2022 Oct 25;14(21):4484. doi: 10.3390/nu14214484 (PMC9654270; doi:10.3390/nu14214484)
Supplement: Supplementary file 1 [file nutrients-14-04484-s001.zip › nutrients-1950842-supplementary.pdf]

Supplementary Material for

The interactive effects of severe vitamin D deficiency and iodine nutrition status on the risk of thyroid disorder in pregnant women

Wei Lu[1], Zhengyuan Wang[1], Zhuo Sun[1], Zehuan Shi[1], Qi Song[1], Xueying Cui[1], Liping Shen[1], Mengying Qu[1], Shupeng Mai[1] and Jiajie Zang *

1 Division of Health Risk Factors Monitoring and Control, Shanghai Municipal Center for Disease Control and Prevention, Shanghai 200336, China;
*Correspondence: zangjiajie@scdc.sh.cn (J.Z.) ; Tel. : +86 - 02162758710

Table S1. Interaction between low UIC and vitamin D deficiency on the risk of hypothyroidism/TgAb/TPOAb/TrAb positive in the first trimester

**Vitamin D (ng/ml)**	**UIC: 150 – 249 ug/L**		**UIC < 150 ug/L**		**OR for UIC within category of vitamin D**	**RERI (95%CI)**	**AP (95%CI)**
	**Hypothyroidism/Normal(N)**	**OR (95%CI)**	**Hypothyroidism/Normal(N)**	**OR (95%CI)**			
⩾ 12	45/288	1	80/544	1.08(0.73, 1.61)	1.08(0.73, 1.61)	-0.352(-1.440, 0.735)	-0.499(-2.15, 1.15)
< 12	8/44	1.10(0.48, 2.52)	10/91	0.69(0.33, 1.44)	1.53(0.53, 4.37)	-	-
OR for vitamin D deficiency within category of UIC	-	1.10(0.48, 2.52)	-	0.74(0.37, 1.49)	-	-	-
	TgAb positive /Normal(N)	OR (95%CI)	TgAb positive /Normal(N)	OR (95%CI)			
⩾ 12	17/316	1	48/576	0.66(0.37, 1.17)	0.66(0.37, 1.17)	0.652(-1.065, 2.370)	0.345(-0.437, 1.128)
< 12	2/50	0.66(0.15, 2.96)	9/92	1.79(0.76, 4.18)	0.41(0.08, 2.00)	-	-
OR for vitamin D deficiency within category of UIC	-	0.66(0.15, 2.96)	-	1.25(0.59, 2.66)	-	-	-
	TPOAb positive /Normal(N)	OR (95%CI)	TPOAb positive /Normal(N)	OR (95%CI)			
⩾ 12	73/260	1	148/476	0.91(0.66, 1.26)	0.91(0.66, 1.26)	0.072(-0.858, 1.001)	0.061(-0.714, 0.835)
< 12	11/41	0.97(0.47, 1.99)	26/75	1.17(0.69, 1.99)	0.75(0.32, 1.74)	-	-
OR for vitamin D deficiency within category of UIC	-	0.97(0.47, 1.99)	-	1.03(0.63, 1.68)	-	-	-
	TrAb positive /Normal(N)	OR (95%CI)	TrAb positive /Normal(N)	OR (95%CI)			
⩾ 12	68/265	1	151/473	0.78(0.56, 1.08)	0.78(0.56, 1.08)	0.128(-0.822, 1.078)	0.100(-0.604, 0.800)
< 12	9/43	0.84(0.39, 1.83)	25/76	1.27(0.74, 2.15)	0.60(0.24, 1.46)	-	-
OR for vitamin D deficiency within category of UIC	-	0.84(0.39, 1.83)	-	1.05(0.64, 1.71)	-	-	-

Table S2. Interaction between high UIC and vitamin D deficiency on the risk of hypothyroidism/TgAb/TPOAb/TrAb positive in the firster trimester

**Vitamin D (ng/ml)**	**UIC: 150 – 249 ug/L**		**UIC ⩾ 249 ug/L**		**OR for UIC within category of vitamin D**	**RERI (95%CI)**	**AP (95%CI)**
	**Hypothyroidism/Normal(N)**	**OR (95%CI)**	**Hypothyroidism/Normal(N)**	**OR (95%CI)**			
⩾ 12	45/288	1	31/249	0.80(0.49, 1.31)	0.80(0.49, 1.31)	0.434(-1.140, 2.008)	0.287(-0.614, 1.189)
< 12	8/44	1.10(0.48, 2.52)	8/35	1.38(0.59, 3.19)	1.61(0.50, 5.21)	-	-
OR for vitamin D deficiency within category of UIC	-	1.10(0.48, 2.52)	-	1.90(0.79, 4.58)	-	-	-
	TgAb positive /Normal(N)	OR (95%CI)	TgAb positive /Normal(N)	OR (95%CI)			
⩾ 12	17/316	1	16/264	1.03(0.50, 2.09)	1.03(0.50, 2.09)	-	-
< 12	2/50	0.66(0.15, 2.96)	0/43	-	-	-	-
OR for vitamin D deficiency within category of UIC	-	0.66(0.15, 2.96)	-	-	-	-	-
	TPOAb positive /Normal(N)	OR (95%CI)	TPOAb positive /Normal(N)	OR (95%CI)			
⩾ 12	73/260	1	73/207	1.21(0.83, 1.76)	1.21(0.83, 1.76)	-0.139(-1.288, 1.009)	-0.127(-1.232, 0.978)
< 12	11/41	0.97(0.47, 1.99)	10/33	1.10(0.51, 2.36)	1.08(0.37, 3.12)	-	-
OR for vitamin D deficiency within category of UIC	-	0.97(0.47, 1.99)	-	0.84(0.39, 1.83)	-	-	-
	TrAb positive /Normal(N)	OR (95%CI)	TrAb positive /Normal(N)	OR (95%CI)			
⩾ 12	68/265	1	55/225	0.94(0.63, 1.41)	0.94(0.63, 1.41)	**1.910(0.054, 3.766)**	**0.700(0.367, 1.032)**
< 12	9/43	0.84(0.39, 1.83)	17/26	**2.62(1.32, 5.22)**	**3.55(1.32, 9.54)**	-	-
OR for vitamin D deficiency within category of UIC	-	0.84(0.39, 1.83)	-	**2.66(1.33, 5.32)**	-	-	-

Bold indicates statistical significance after adjustment for age, BMI, annual household income, educational level, gestation week, season, smoking and drinking status as well as vitamin D intake

Table S3. Interaction between low UIC and vitamin D deficiency on the risk of hypothyroidism/TgAb/TPOAb/TrAb positive in the second trimester

**Vitamin D (ng/ml)**	**UIC: 150 - 249 ug/L**		**UIC < 150 ug/L**		**OR for UIC within category of vitamin D**	**RERI (95%CI)**	**AP (95%CI)**
	**Hypothyroidism/Normal(N)**	**OR (95%CI)**	**Hypothyroidism/Normal(N)**	**OR (95%CI)**			
⩾ 12	35/321	1	71/658	0.98(0.64, 1.51)	0.98(0.64, 1.51)	0.804(-0.261, 1.871)	0.575(-0.043, 1.195)
< 12	3/48	0.62(0.18, 2.12)	14/94	1.38(0.71, 2.69)	0.32(0.08, 1.29)	-	-
OR for vitamin D deficiency within category of UIC	-	0.62(0.18, 2.12)	-	1.35(0.73, 2.51)	-	-	-
	TgAb positive /Normal(N)	OR (95%CI)	TgAb positive /Normal(N)	OR (95%CI)			
⩾ 12	17/339	1	29/700	1.27(0.69, 2.36)	1.27(0.69, 2.36)	-	-
< 12	0/51	-	6/102	1.14(0.43, 2.98)	-	-	-
OR for vitamin D deficiency within category of UIC	-	-	-	1.49(0.60, 3.70)		-	-
	TPOAb positive /Normal(N)	OR (95%CI)	TPOAb positive /Normal(N)	OR (95%CI)			
⩾ 12	74/282	1	120/609	**1.39(1.01, 1.93)**	**1.39(1.01, 1.93)**	0.085(-0.780, 0.949)	0.096(-0.872, 1.064)
< 12	12/39	1.16(0.57, 2.37)	21/87	0.90(0.52, 1.56)	1.36(0.59, 3.09)	-	-
OR for vitamin D deficiency within category of UIC	-	1.16(0.57, 2.37)	-	1.24(0.74, 2.09)	-	-	-
	TrAb positive /Normal(N)	OR (95%CI)	TrAb positive /Normal(N)	OR (95%CI)			
⩾ 12	86/270	1	145/584	1.30(0.95, 1.76)	1.30(0.95, 1.76)	0.029(-0.918,0.977)	0.027(-0.845, 0.902)
< 12	15/36	1.24(0.64, 2.41)	28/80	1.09(0.66, 1.79)	1.35(0.62, 2.91)	-	-
OR for vitamin D deficiency within category of UIC	-	1.24(0.64, 2.41)	-	1.41(0.88, 2.27)	-	-	-

Table S4. Interaction between high UIC and vitamin D deficiency on the risk of hypothyroidism/TgAb/TPOAb/TrAb positive in the second trimester

Vitamin D (ng/ml)	UIC: 150 – 249 ug/L		UIC ⩾ 249 ug/L		OR for UIC within category of vitamin D	RERI (95%CI)	AP (95%CI)
	Hypothyroidism/Normal(N)	OR (95%CI)	Hypothyroidism/Normal(N)	OR (95%CI)			
≥ 12	35/321	1	28/209	1.17(0.69, 1.99)	1.17(0.69, 1.99)	-0.030(-1.243, 1.183)	-0.044(-1.820, 1.733)
< 12	3/48	0.62(0.18, 2.12)	3/38	0.75(0.21, 2.66)	1.74(0.27, 11.23)	-	-
OR for vitamin D deficiency within category of UIC	-	0.62(0.18, 2.12)	-	0.54(0.16, 1.91)	-	-	-
	TgAb positive /Normal(N)	OR (95%CI)	TgAb positive /Normal(N)	OR (95%CI)			
≥ 12	17/339	1	8/229	0.76(0.32, 1.80)	0.76(0.32, 1.80)	-	-
< 12	0/51	-	5/36	2.94(0.98, 8.84)	-	-	-
OR for vitamin D deficiency within category of UIC		-	-	**3.53(1.14, 11.98)**	-	-	-
	TPOAb positive /Normal(N)	OR (95%CI)	TPOAb positive /Normal(N)	OR (95%CI)			
≥ 12	74/282	1	41/196	0.78(0.50, 1.20)	0.78(0.50, 1.20)	0.244(-0.874, 1.369)	0.222(-0.708, 1.15)
< 12	12/39	1.16(0.57, 2.37)	11/30	1.15(0.54, 2.45)	0.96(0.35, 2.59)	-	-
OR for vitamin D deficiency within category of UIC	-	1.16(0.57, 2.37)	-	1.42(0.64, 3.15)	-	-	-
	TrAb positive /Normal(N)	OR (95%CI)	TrAb positive /Normal(N)	OR (95%CI)			
≥ 12	86/270	1	48/189	0.77(0.52, 1.16)	0.77(0.52, 1.16)	-0.064(-1.161, 1.033)	-0.066(-1.228, 1.095)
< 12	15/36	1.24(0.64, 2.41)	10/31	0.95(0.44, 2.05)	0.71(0.27, 1.8)	-	-
OR for vitamin D deficiency within category of UIC	-	1.24(0.64, 2.41)	-	1.27(0.57, 2.80)	-	-	-

Bold indicates statistical significance after adjustment for age, BMI, annual household income, educational level, gestation week, season, smoking and drinking status as well as vitamin D intake

Table S5. Interaction between low UIC and vitamin D deficiency on the risk of hypothyroidism/TgAb/TPOAb/TrAb positive in the third trimester

**Vitamin D (ng/ml)**	**UIC: 150 – 249 ug/L**		**UIC < 150 ug/L**		**OR for UIC within category of vitamin D**	**RERI (95%CI)**	**AP (95%CI)**
	**Hypothyroidism/Normal(N)**	**OR (95%CI)**	**Hypothyroidism/Normal(N)**	**OR (95%CI)**			
⩾ 12	43/232	1	97/619	1.20(0.81, 1.78)	1.20(0.81, 1.78)	-0.587(-1.708, 0.535)	-1.414(-4.452, 1.624)
< 12	8/35	1.20(0.51, 2.83)	7/86	0.42(0.18, 1.00)	2.63(0.80, 8.65)	-	-
OR for vitamin D deficiency within category of UIC	-	1.20(0.51, 2.83)	-	0.52(0.23, 1.16)	-	-	-
	TgAb positive /Normal(N)	OR (95%CI)	TgAb positive /Normal(N)	OR (95%CI)			
⩾ 12	3/272	1	18/698	0.42(0.12, 1.46)	0.42(0.12, 1.46)	-	-
< 12	0/43	-	2/91	1.83(0.29, 11.61)	-	-	-
OR for vitamin D deficiency within category of UIC	-	-	-	0.89(0.20, 4.02)	-	-	-
	TPOAb positive /Normal(N)	OR (95%CI)	TPOAb positive /Normal(N)	OR (95%CI)			
⩾ 12	41/234	1	142/574	0.71(0.48, 1.03)	0.71(0.48, 1.03)	-0.042(-0.924, 0.841)	-0.048(-1.08, 0.987)
< 12	3/40	0.39(0.11, 1.37)	12/81	0.87(0.43, 1.74)	0.50(0.13, 1.94)	-	-
OR for vitamin D deficiency within category of UIC	-	0.39(0.11, 1.37)	-	0.59(0.31, 1.12)	-	-	-
	TrAb positive /Normal(N)	OR (95%CI)	TrAb positive /Normal(N)	OR (95%CI)			
⩾ 12	47/228	**1**	156/560	0.74(0.51, 1.06)	0.74(0.51, 1.06)	0.859(-0.214, 1.932)	**0.465(0.005, 0.924)**
< 12	5/38	0.57(0.21, 1.57)	25/68	1.75(0.99, 3.08)	**0.26(0.08, 0.82)**	-	-
OR for vitamin D deficiency within category of UIC	-	0.57(0.21, 1.57)	-	1.34(0.81, 2.20)	-	-	-

Bold indicates statistical significance after adjustment for age, BMI, annual household income, educational level, gestation week, season, smoking and drinking status as well as vitamin D intake

Table S6. Interaction between high UIC and vitamin D deficiency on the risk of hypothyroidism/TgAb/TPOAb/TrAb positive in the third trimester

**Vitamin D (ng/ml)**	**UIC: 150 – 249 ug/L**		**UIC ⩾ 249 ug/L**		**OR for UIC within category of vitamin D**	**RERI (95%CI)**	**AP (95%CI)**
	**Hypothyroidism/Normal(N)**	**OR (95%CI)**	**Hypothyroidism/Normal(N)**	**OR (95%CI)**			
⩾ 12	43/232	1	27/151	0.92(0.54, 1.58)	0.92(0.54, 1.58)	1.238(-1.226, 3.702)	0.534(-0.105, 1.174)
< 12	8/35	1.20(0.51, 2.83)	6/13	2.43(0.86, 6.89)	1.79(0.47, 6.85)	-	-
OR for vitamin D deficiency within category of UIC	-	1.20(0.51, 2.83)	-	2.66(0.92, 7.71)	-	-	-
	Tgab positive /Normal(N)	OR (95%CI)	Tgab positive /Normal(N)	OR (95%CI)			
⩾ 12	3/272	1	9/169	**4.02(1.04, 15.61)**	**4.02(1.04, 15.61)**	-	-
< 12	0/43	-	0/19	-	-	-	-
OR for vitamin D deficiency within category of UIC	-	-	-	-	-	-	-
	TPOAb positive /Normal(N)	OR (95%CI)	TPOAb positive /Normal(N)	OR (95%CI)			
⩾ 12	41/234	1	34/144	1.24(0.74, 2.07)	1.24(0.74, 2.07)	0.791(-0.956, 2.538)	0.561(-0.177, 1.298)
< 12	3/40	0.39(0.11, 1.37)	4/15	1.39(0.42, 4.69)	3.63(0.66, 20.08)	-	-
OR for vitamin D deficiency within category of UIC	-	0.39(0.11, 1.37)	-	1.32(0.39, 4.47)	-	-	-
	Trab positive /Normal(N)	OR (95%CI)	Trab positive /Normal(N)	OR (95%CI)			
⩾ 12	47/228	**1**	31/147	0.95(0.57, 1.58)	0.95(0.57, 1.58)	2.123(-0.451, 4.697)	**0.829(0.512, 1.146)**
< 12	5/38	0.57(0.21, 1.57)	7/12	2.70(0.96, 7.54)	**5.95(1.31, 27.05)**	-	-
OR for vitamin D deficiency within category of UIC	-	0.57(0.21, 1.57)	-	**3.22(1.10, 9.45)**	-	-	-

Bold indicates statistical significance after adjustment for age, BMI, annual household income, educational level, gestation week, season, smoking and drinking status as well as vitamin D intake
